# Rhinoconjunctival sensitization to hydrolyzed wheat protein in facial soap and induce wheat-dependant exercise-induced anaphylaxis

**DOI:** 10.1186/2045-7022-1-S1-P49

**Published:** 2011-08-12

**Authors:** Yuma Fukutomi, Yasuharu Itagaki, Masami Taniguchi, Akemi Saito, Hiroshi Yasueda, Takuya Nakazawa, Maki Hasegawa, Hiroyuki Nakamura, Kazuo Akiyama

**Affiliations:** 1 Sagamihara National Hospital, Clinical Research Center for Allergy and Rheumatology, Sagamihara Kanagawa, Japan; 2 Hokkaido Bunkyo University, Department of Health and Nutrition, Faculty of Human Science, Eniwa, Japan; 3 Graduate School of Medical Science, Kanazawa University, Kanazawa Ishikawa, Japan; 4 Sagamihara National Hospital, Sagamihara, Clinical Research Center for Allergy and Rheumatology, Sagamihara Kanagawa, Japan

## Background

Wheat protein is responsible for various kinds of allergic disease. Wheat-dependent exercise-induced anaphylaxis (WDEIA) is one of the most important clinical phenotypes of adult wheat allergy. Relatively homogenous clinical and immunological presentations of this phenotype have already been described. More recently, some studies have shown that sensitization to hydrolyzed wheat protein (HWP) in cosmetics can induce allergy to HWP-containing food as well as contact allergy to HWP-containing cosmetics.

## Case reports

We experienced treating five Japanese women with WDEIA with atypical presentation. They presented exercise-induced anaphylaxis after ingestion of normal wheat products, but also had episodes of skin and/or rhinoconjunctival contact allergy to hydrolyzed wheat protein (HWP)-containing facial soap. Furthermore, case histories and serological analyses of these patients indicated that the development of their WDEIA was induced by primary sensitization to HWP in the facial soap they used and accompanying sensitization to natural wheat protein.

## Methods and results

All of the patients had started to use the same facial soap product containing HWP 1 to 3 years prior to the onset of WDEIA. At first, they used the facial soap without any problem. When they continued to use the soap, they started to experience itchiness and urticaria of the eyelids or face after using the soap. Their symptoms of allergy to facial soap resembled prodromal symptoms that develop following the combination of wheat ingestion and exercise. A significantly higher IgE reactivity to HWP than to natural wheat protein was observed in these patients. Inhibition analyses of their sera showed that IgE reactivity to HWP was not inhibited by natural wheat extracts, whereas those to natural wheat extracts were fully inhibited by HWP, indicating that the primary sensitizer of these patients is HWP.

## Discussion

Our report is important in that it indicates the possible role of HWP included in cosmetics in the induction of allergy to natural wheat products.

